# Correlation of T cell response and bacterial clearance in human volunteers challenged with *Helicobacter pylori* revealed by randomised controlled vaccination with Ty21a-based *Salmonella* vaccines

**DOI:** 10.1136/gut.2007.145839

**Published:** 2008-04-16

**Authors:** T Aebischer, D Bumann, H J Epple, W Metzger, T Schneider, G Cherepnev, A K Walduck, D Kunkel, V Moos, C Loddenkemper, I Jiadze, M Panasyuk, M Stolte, D Y Graham, M Zeitz, T F Meyer

**Affiliations:** 1 Department of Molecular Biology, Max Planck Institute for Infection Biology, Berlin, Germany; 2 Medical Clinic I, Charite Campus Benjamin Franklin, Berlin, Germany; 3 Institute for Pathology, Charite Campus Benjamin Franklin, Berlin, Germany; 4 Kazan State University, Kazan, Russia; 5 Institute for Pathology, Klinikum Bayreuth, Bayreuth, Germany; 6 Veterans Affairs Medical Center Houston, Texas, USA

## Abstract

**Background:**

*Helicobacter pylori* remains a global health hazard, and vaccination would be ideal for its control. Natural infection appears not to induce protective immunity. Thus, the feasibility of a vaccine for humans is doubtful.

**Methods:**

In two prospective, randomised, double-blind, controlled studies (Paul Ehrlich Institute application nos 0802/02 and 1097/01), live vaccines against *H pylori* were tested in human volunteers seronegative for, and without evidence of, active *H pylori* infection. Volunteers (n = 58) were immunised orally with *Salmonella enterica* serovar Typhi Ty21a expressing *H pylori* urease or HP0231, or solely with Ty21a, and then challenged with 2×10^5^ cagPAI^−^ *H pylori.* Adverse events, infection, humoral, cellular and mucosal immune response were monitored. Gastric biopsies were taken before and after vaccination, and postchallenge. Infection was terminated with antibiotics.

**Results:**

Vaccines were well tolerated. Challenge infection induced transient, mild to moderate dyspeptic symptoms, and histological and transcriptional changes in the mucosa known from chronic infection. Vaccines did not show satisfactory protection. However, 13 of 58 volunteers, 8 vaccinees and 5 controls, became breath test negative and either cleared *H pylori* (5/13) completely or reduced the *H pylori* burden (8/13). *H pylori*-specific T helper cells were detected in 9 of these 13 (69%), but only in 6 of 45 (13%) breath test-positive volunteers (p = 0.0002; Fisher exact test). T cells were either vaccine induced or pre-existing, depending on the volunteer.

**Conclusion:**

Challenge infection offers a controlled model for vaccine testing. Importantly, it revealed evidence for T cell-mediated immunity against *H pylori* infection in humans.

Half of the world’s population is infected with *Helicobacter pylori.* Infection causes gastritis, peptic ulcer and gastric cancer.^1^ Although infection may be beneficial in some cases,^2^ ^3^ its pathological consequences outweigh currently projected beneficial roles. Antibiotic resistance and compliance problems significantly reduce treatment efficacy.^4^ In developing countries, re-infection is common, and current treatment options are inadequate for control. An effective vaccine, however, could prevent re-infection and offer cost-effective infection management.

To date, immunity against *H pylori* has only been obtained in animal models^5^ ^6^ where protection depends, at least in part, on induction of T helper cells.^7^ In contrast, natural infection in humans appears specifically to inhibit T cell responses via induction of regulatory T cells^8^ ^9^ and direct inhibition of T cell activation.^10^^–^12 Although various vaccines have been tested in clinical trials (for reviews see Ruggiero *et al*.^13^ and Aebischer *et al*^14^), it remains unclear whether immunity against *H pylori* exists in humans and whether vaccination is feasible.

We tested live vaccines based on recombinant *Salmonella* Ty21a, the licensed typhoid fever vaccine, in volunteers subsequently challenged with *H pylori*.^15^ Although the vaccines were not satisfactory, the studies revealed clearly that T cell reactivity against *H pylori* antigens correlated with clearance or significant reduction of *H pylori* burden.

## METHODS

### Vaccine strain construction and challenge *H pylori* strain

Construction of recombinant Ty21a vaccines expressing *H pylori* urease A and B subunits (Ty21a(pUreA/B)) or HP0231 (Ty21a(pHP0231), respectively, is described in the supplementary material and in Bumann *et al*.^16^ Frozen *Salmonella* of known 10^10^ colony-forming units (cfu) were thawed and resuspended in 30 ml of phosphate-buffered saline (PBS) for oral vaccination.

The *H pylori* challenge strain has been described.^15^ Volunteers were infected with 2×10^5^ freshly grown bacteria, resuspended in 30 ml of instant soup (Maggi) for infection. Cfus were confirmed by plating.

### Study design

Two prospective, randomised, double-blind, controlled studies were designed (for diagrams see supplementary figs 2 and 3) as a combination of two consecutive protocols: vaccination and challenge infection. Infection was monitored over predefined periods before the application of antibiotic therapy to terminate infection, independent of vaccine effect. The first study was planned as a pilot trial to assess primarily the safety of the approach. The second study was planned based on the outcome of the first trial to assess protective effects.

### Participants

The pilot study was conducted in 2003–4. Twenty subjects were enrolled at the Medical Clinic I, Charité Campus Benjamin Franklin, Berlin. Eligible subjects were male, aged between 20 and 50 years, healthy with normal routine blood and chemical laboratory parameters, negative for *H pylori* infection by [13C]urea breath test (UBT), serology and stool antigen test. Exclusion criteria were abnormal upper gastrointestinal tract endoscopy including analysis of biopsies, history of typhoid fever vaccination, history of disease of the biliary or gastrointestinal tract, recent drug prescriptions, allergies to antibiotics, a diet rich in sour or fermented food, cases of gastric cancer in close relatives and regular contact with children younger than 12 years.

The second study was conducted between December 2004 and April 2006. Of 133 subjects assessed and screened as above, 47 volunteers were enrolled at the Medical Clinic I, Charité, Campus Benjamin Franklin, Berlin.

### Ethics

The study protocols were developed adhering to the Declaration of Helsinki, reviewed and approved by the ethical review board of the Charité, Berlin, and the studies were registered with the responsible German federal authority, the Paul Ehrlich Institute (applications nos 0802/02 and 1097/01).

All volunteers were informed about the study protocol, potential risks and adverse reactions to vaccination and challenge infection before giving written consent.

### Interventions

In the first study, there were three treatment arms for vaccination: volunteers were given three doses of 10^10^ bacteria orally on day 0, 2 and 4 of the Ty21a control (n = 4), or Ty21a(pUreA/B) vaccines containing either expression plasmid pDB2 (n = 8) or pDB3 (n = 8). On day 5, 7, 10 and 28 postvaccination, blood was taken for serology and analysis of responses in peripheral blood monocytic cells (PBMCs). Four weeks after vaccination, gastroscopy with biopsy was repeated. Volunteers were interviewed repeatedly to record untoward events.

Because serious adverse events due to challenge could not be excluded, the code was broken in week 6 after vaccination to select one volunteer per treatment arm (volunteer nos 2, 8 and 16) based on a similar response to the Ty21a carrier. Volunteers remained blinded and were challenged with *H pylori* on day 42 postvaccination and again alerted to report adverse reactions. Infection was monitored by UBT on day 3, 7, 14 and 38 post-challenge and weekly thereafter. Serum was obtained on day 7 and 14, and PBMCs were obtained on day 38 postchallenge. Endoscopy was repeated in week 6 after challenge, and biopsies were taken for *H pylori* culture, histology, determination of local cytokine secretion and mRNA preparation. Thereafter, *H pylori* was eradicated by triple therapy for 7 days combining amoxicillin, clarithromycin and pantoprazol (ACP; ZacPac). Eradication was confirmed by UBT on days 68 and 132 after challenge. As gastritis was similar in all three subjects and no severe untoward events were noted, the remaining 17 volunteers were contacted. One could not be contacted and six volunteers declined. Ten volunteers were enrolled for challenge infection which took place 5 months after vaccination. Monitoring and sample collection were performed at the same time intervals postchallenge as before. Therapy was initiated in week 8 instead of week 6 postchallenge.

In the second study volunteers were allocated to a first arm for vaccination with control Ty21a (n = 22). Volunteers allocated to a second arm (n = 12) were given the recombinant Ty21a(pUreA/B) holding plasmid pDB2 used in the first study. Volunteers in the third arm (n = 13) were given a novel vaccine strain expressing antigen HP0231, Ty21a(pHP0231). Given capacity constraints, the rationale for introducing the latter and omitting one Ty21a(pUreA/B) vaccine used in the first study was as follows: no differences in immunogenicity were detected between Ty21a(pUreA/B) vaccines; hence only one was selected for further testing. Ty21a(pHP0231) was included because corresponding vaccines were equally efficient in protecting mice compared with the gold standards *H pylori* lysate and *S typhimurium* (pureA/B)^22^ (TA, DB unpublished). Furthermore, the North American schedule of four vaccine doses given every other day was adopted to maximise responder numbers.

Of the 47 vaccinated volunteers, 45 were infected with *H pylori* on day 42 as described above. Two withdrew from the study. The observation period after challenge infection was extended to 3 months and instead of blood collection and a gastroscopy on day 28 (postvaccination), blood samples were collected and gastroscopy performed after week 6 and 10 postinfection. Infection was terminated by antibiotic therapy as described above, and success was monitored 1 and 3 months later.

### Objectives

The primary objectives were to assess safety, immunogenicity and potential protective effect of prophylactic vaccination with live, recombinant *Salmonella* Ty21a vaccines in human volunteers experimentally challenged with *H pylori*. The secondary objective was to test whether protection, if it existed, was correlated with a particular type of immune response.

### Outcomes

The primary end point was protection from a *H pylori* challenge—that is, negative testing in four diagnostic tests for active *H pylori* infection. The four tests were UBT, rapid urease test (RUT) on biopsies, *H pylori* detection by histochemistry and culture from biopsies.

Secondary outcomes were histological changes and T and B cell response patterns, assessed by cytokine and antibody production, respectively. A secondary end point for evidence of protection was re-defined for post hoc analyses and consisted of repeatedly negative UBT and negativity in at least one of the three remaining diagnostic tests.

### Sample size

The first study was designed as a pilot study primarily to assess the safety of the vaccination cum challenge infection model. Sample size was determined based on previous studies with the vaccines used, when 33–56% of volunteers generated a specific T cell response. Thus, groups of 8 volunteers were immunised with the recombinant vaccines and 4 volunteers were given the control Ty21a. It was expected that 30–50% of volunteers would generate a urease-specific T cell response and, if this were correlated with protection, the trend should be detectable.

A minimal group size of 20 in the vaccine (cumulated vaccine groups) and 20 in the control groups for the second study was determined using the PS program (http://biostat.mc.vanderbilt.edu/twiki/bin/view/Main/PowerSampleSize) based on the outcome of the first study—that is, indications of clearing response in 5 of 9 vaccinees (55%) and assuming a frequency of 10% of volunteers in the control groups with a clearing response. The hypothesis that no difference between vaccinees and controls existed was to be tested with a power of 0.8 at a significance level of 0.05 by two-sided Fisher exact test using a dichotomous outcome (protected/non-protected).

### Randomisation

Volunteers were assigned a continuous number upon screening that served as a volunteer ID throughout the studies. To generate a random sequence of vaccine allocation, ID numbers of enrolled volunteers were drawn blindly from a hat by a collaborator not involved in screening or enrolment. The allocation key was concealed and not disclosed to personnel involved in administering the vaccines.

On the days of vaccination, volunteers were given vaccines in ascending order according to their ID, being administered vaccine doses labelled with their ID by the person who generated the allocation key.

The study physician and personnel involved in direct contact with volunteers were blinded and ignorant of the allocation sequence of treatment arms.

### Analytical procedures

Histological analyses were performed as described^17^ by a single pathologist (MS) blinded to treatment keys.

Antibody-secreting cells (ASCs) and specific T cells were monitored. Cryopreserved PBMCs from the same volunteer collected at different times were thawed and analysed simultaneously. ASCs were enumerated by ELISPOT.^18^ T cells were analysed by multichromatic cytofluorimetry^19^ ^20^ after in vitro stimulation of PBMCs with a *Salmonella* lysate (anticarrier response), and with recombinant urease A (first study) or overlapping peptide pools (15mer peptides with 12mer overlaps) representing urease A/B or HP0231 (see supplementary information). Cells cultured in medium or stimulated with Staphylococcal enterotoxin B served as negative and positive controls, respectively. Responses were compared in PBMCs prepared before vaccination, at day 5 postvaccination, and 6 weeks and 3 months after challenge infection, totalling >1000 samples.

Local cytokines were determined in biopsy culture supernatants^21^ by cytometric bead array (CBA system, BD; see supplementary information).

Transcriptional profiling was performed analysing total RNA from antrum biopsies on a custom-made microarray (see supplementary information).

### Statistical analyses

Quantitative parameters of post hoc analyses are presented as medians and range when data sets were not all normally distributed, and groups compared by two-sided, non-parametric tests using Graphpad Prism4.

Significant T cell responses were determined based on a threshold of mean prevaccination level +3 SD after testing that prevaccination numbers of memory CD45RA^−^, α4β7 integrin^+^ CD4^+^ cells expressing interferon γ (IFNγ) (first study) or CD40L and interleukin 2 (IL2) (second study) were normally distributed (identified outliers in study 2 were excluded from analysis). Volunteers were classified into CD4 T cell responders and non-responders and, based on UBT results, as UBT^+^ or UBT^−^. As a null hypothesis, the independence of UBT results and CD4 T cell responses was evaluated by two-sided Fisher exact test.

Differences in T cell response quality between UBT^+^ and UBT^−^ groups were analysed using Statistica 6 software by factorial analysis of data on 14 different T cell subpopulations (eg, CD4^+^ or CD8^+^, CD40L^+^ or CD40L ^−^, IFNγ^+^ or IFNγ^−^ cells analysed at four time points and restimulated in vitro with different antigens). The first factor captured >70% of the variation in either group and was composed of different T cell subpopulations.

## RESULTS

### Adverse effects of vaccination and challenge infection

Vaccines were well tolerated, with minor adverse effects such as gastrointestinal nausea (2/20), mild diarrhoea (2/20) and headaches (5/20) reported on single days over a 28 day period (see supplementary table 1). Infection with *H pylori* caused dyspeptic symptoms such as nausea and abdominal pain that started around day 3 after challenge, but were transient and resolved after 2 weeks in the great majority of volunteers (supplementary fig 2). In each treatment group, one volunteer developed transient grade 3 adverse effects after challenge, with nausea, vomiting, strong abdominal pain and diarrhoea. One volunteer developed a small ventricular ulcer that had completely resolved after 2 weeks of proton pump inhibitor therapy. No serious adverse events were observed, and neither vaccination nor challenge interfered significantly with the volunteers’ daily activities.

**Table 1:** Effects of vaccination on *H pylori* infection (first study)

Vaccine	Ty21a control				Ty21a(pUreA/B)								
Volunteer	2	6	7	12	4	8*	10	13*	15	5*	16	18	20
Test†													
*H pylori* culture	–	–	+	+	+	–	+	–	+	–	–	–	–
RUT	+	–	+	+	+	–	+	–	+	–	–	–	**+**
UBT	+	–	+	+	+	+	+	+	+	–	–	–	–
*H pylori* histology	+	**+**	–	+	+	+	+	+	+	–	–	–	–
Score‡	3	**1**	3	4	4	**2**	4	**2**	4	**0**	**0**	**0**	**1**

*Volunteers receiving the Ty21a(pureA/B) vaccine containing plasmid pDB3.

†*H pylori* colonisation was determined by culture of biopsies, RUT on biopsies and Warthin Starry staining of biopsy sections in addition to UBT.

‡Sum of positive tests at 6 weeks and/or 3 months postchallenge; clearing volunteers with a score ⩽2 are marked in bold.

RUT, rapid urease test; UBT, [13C]urea breath test.

### Course of infection in vaccinated and control-treated volunteers

Infection was monitored by four tests: continuously by UBT, and by RUT, silver staining and culture of biopsy material obtained after 6 weeks and in the third month of infection (second study only). Test results were classified as positive or negative to compute an overall *H pylori* score (0 = all tests negative, 4 = all positive). Volunteers tested negative in all tests at baseline. Protection as a primary end point was defined as a *H pylori* score of 0 in week 6.

**Figure 1:**
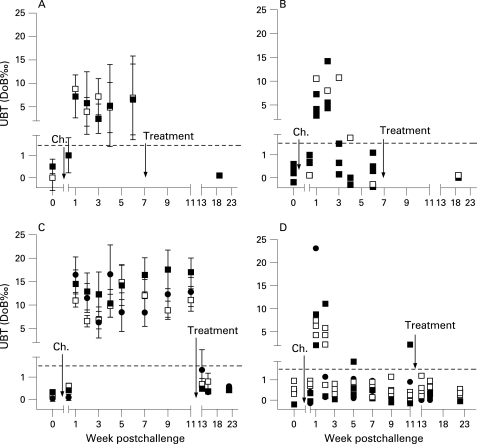
Course of *H pylori* infection in vaccinated volunteers. A breath test was used to monitor the course of infection in experimentally challenged volunteers over time. (A, B) [13C]urea breath test (UBT) results of 4 Ty21a control (□) and 9 Ty21a(pUreA/B) (▪) vaccinated volunteers in the first study. (C, D) UBT results of control (□), Ty21a(pUreA/B) (▪) and Ty21a(pHP0231) (•) vaccinated volunteers in the second study. Values correspond to mean (SD) UBT results of vaccine groups repeatedly tested at the indicated times postchallenge infection. (B) and (D) UBT results over time of individual volunteers in study 1 and 2, respectively, who although initially testing positive after challenge, tested repeatedly negative (threshold UBT values DoB <1.5; stippled line) after week 3. ‘Ch.’ and arrow denote time of challenge infection, ‘Treatment’ and arrow mark the start time of antibiotic eradication.DoB, delta over baseline.

In the first study, which was designed as a pilot trial, 13 of 20 volunteers vaccinated were infected with *H pylori* (4 controls and 9 vaccinees) and all developed a positive UBT by day 7 after challenge, indicating reliable and successful infection (fig 1A). Five individuals developed repeatedly negative UBT after week 3 postchallenge (fig 1B). In week 6, three of nine vaccinees (33%) but none of the four control volunteers had a score of 0 (table 1), suggesting a protective vaccine effect. Therefore, protection was tested in a second study.

The second study followed the same design principle to evaluate the live vaccines Ty21a(pureA/B) used in the first trial and a novel vaccine, Ty21a(pHP0231) expressing the *H pylori* antigen HP0231 that was found in a mouse model to be protective.^22^ Disappointingly, there was no significant difference between vaccine and control groups with regard to the primary end point, *H pylori* infection (table 2).

**Table 2:** Effects of vaccination on *H pylori* infection (second study)

	Volunteer																																																				
Vaccine Ty21a control																																																					
	8	13	18	23			29			71			77		81		90			96		98			101			102			103			104		108		121	125			127			129						132		
Test*																																																					
*H pylori* culture	+	+	–	–			+			+			+		+		–			+		+			+			+			+			–		+		–	+			+			–						**+**		
RUT	+	+	–	+			+			+			–		+		–			–		+			+			+			+			–		–		+	+			+			+						–		
UBT	+	+	–	+			+			+			+		+		–			+		+			+			+			+			–		+		+	+			+			+						–		
*H pylori* histology	+	+	**+**	+			+			+			+		+		**+**			+		+			+			+			+			–		+		+	+			+			+						–		
**Score**	4	4	**1**	3			4			4			3		4		**1**			3		4			4			4			4			**0**		3		3	4			4			3						**1**		
Vaccine Ty21a(pUreA/B)																																																					
Volunteer						5			12		16			39		48			70				72			78			87			99		106		119																	
Test																																																					
*H pylori* culture						+			+		**+**			+		**+**			+				–			+			+			+		+		+																	
RUT						+			+		–			+		–			+				–			+			+			–		+		+																	
UBT						+			+		–			+		–			+				–			+			+			+		+		+																	
*H pylori* histology						+			+		–			+		**+**			+				**+**			+			+			+		–		+																	
**Score**						4			4		**1**			4		**2**			4				**1**			4			4			3		3		4																	
Vaccine Ty21a(pHP0231)																																																					
Volunteer					14			19				35			43			46			60			66			73			75			91		107		117																
Test																																																					
*H pylori* culture					+			+				+			+			+			+			+			+			+			+		–		+																
RUT					+			+				+			+			+			+			+			+			+			+		–		+																
UBT					+			+				+			+			+			+			+			+			+			+		–		+																
*H pylori* histology					+			+				+			+			+			+			+			+			+			+		–		+																
**Score**					4			4				4			4			4			4			4			4			4			4		**0**		4																

*For definition of tests and score see the footnotes of table 1.

RUT, rapid urease test; UBT, [13C]urea breath test.

However, 6 weeks after challenge, the *H pylori* score was 0 in two volunteers, and six others were negative in two of four tests. Similarly to the first study, after initially testing positive, these volunteers repeatedly tested negative in UBT after week 3 (fig 1C,D). Four volunteers with a *H pylori* score of <2 were observed in the control group and four in the recombinant vaccine-treated groups. Of note, clearance after successful infection has not been observed in 18 volunteers in previous studies designed to define the infectious dose of the present challenge strain.^15^

### Histological response to vaccination and challenge

Antrum and corpus mucosa were evaluated for the grade of leucocytic infiltrations, infiltrate activity, grade of regenerative epithelial replacement and mucus depletion. These four parameters were individually scored on a scale of 0–3 and scores were cumulated to reflect overall histological changes due to vaccination and infection.

Vaccination alone had no detectable effect on the gastric mucosa (cf. supplementary table 2) and did not induce significant changes in local cytokine production (not shown). In contrast, all volunteers developed typical *H. pylori* gastritis^15^ 6 weeks after challenge. Importantly, vaccination did not exacerbate inflammation (cf. supplementary tables 2 and 3 for first and second study, respectively). Transcriptional profiling of biopsies taken in week 6 showed that infection increased mucosal chemokine and cytokine mRNA abundance (see supplementary table 4 for a list of all affected mRNAs). In agreement with this, we measured higher levels of IL1-β, IL2, IL4, IL5, IL6, IL10, IL12, IFNγ or tumour necrosis factor α (TNFα) protein in supernatants of cultured biopsies from that time point (see supplementary fig 5), yet detected no differences in these between treatment groups. Regulatory T cells expressing the FoxP3 transcription factor^23^ were also enumerated in the mucosa, as these cells are thought to contribute to chronic infection.^8^ ^9^ FoxP3^+^ cells were rare in uninfected stomach mucosa and the 6-week infected organs, but numbers increased 3 months after infection (supplementary fig 6).

### Immunological responses

*Salmonella* vaccination can induce broad, mucosa-targeted B and T cell responses.^24^ ^25^ B cell responses were analysed at day 7 after the first vaccination when ASC numbers against the carrier peak in circulation. Vaccination induced significant numbers of anti-*Salmonella*-specific ASCs in 25% of volunteers in the first study and, depending on the group, in 45–75% in the second study (supplementary table 5). B cell responses to the carrier were in the range expected for these Ty21a vaccines but, as reported,^16^ ^18^ vaccine antigen-specific ASCs were below detection.

In contrast, not only *Salmonella*-carrier but also vaccine antigen-specific—that is, cytokine-producing, eg IFNγ^+^ T cells were detectable by multicolour flow cytometry in in vitro stimulated PBMCs (supplementary table 6). After vaccination (day 5), carrier-specific CD4^+^ T cells were detected in 20–25% of volunteers in both studies. Urease-specific CD4^+^ T cells were observed in 25% of Ty21a(pureA/B) in study 1 and in 8% of the respective vaccinees in study 2. HP0231-specific T cells were detectable in 33% of Ty21a(pHP0231)-vaccinated individuals. Antigen-specific CD8^+^ T cells were not observed (not shown).

After challenge, we recorded urease-specific T cells in 56% of Ty21a(pureA/B) vaccinees (up from 25%) in the first study and 25% (up from 8%) in the second study. In the Ty21a(pHP0231)-treated group, an increase was only observed 3 months after infection (to 42% from 33%).

Unexpectedly, T cells reactive to carrier antigens were detected in five, and against vaccine antigens in six volunteers already before vaccination. In four of the latter, these reactions were against both vaccine antigens, possibly indicating previous exposure to *H pylori.*

### Post hoc analyses

#### Comparative analysis of secondary outcomes in UBT^+^ and UBT^−^ volunteers

While 43 of 58 (74%) volunteers in the two studies remained UBT positive until infections were eradicated, 13 (22%) tested repeatedly negative and were negative in at least one additional diagnostic test. As this may have indicated active reduction of *H pylori* burden, a negative UBT and a *H pylori* score of <2 was used to classify volunteers into UBT^−^ and UBT^+^ groups for a post hoc analysis.

**Figure 2:**
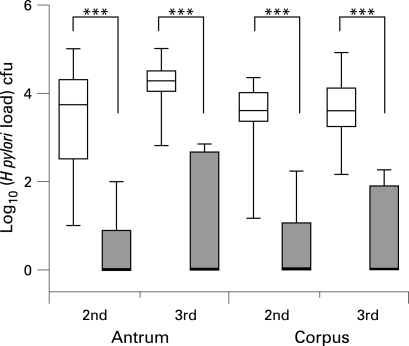
*H pylori* burdens are significantly reduced in [13C]urea breath test (UBT)-negative volunteers. Biopsies obtained 6 weeks (second gastroscopy; 2nd) and 3 months (third gastroscopy; 3rd) after infection from the antrum and corpus were processed to determine *H pylori* colony-forming units (cfu). Data are represented by box and whisker plots which indicate median, range and 25th–75th centiles of the log_10_ transform of cfu counted per biopsy of the UBT*^−^* (shaded box) and UBT^+^ (open box) group of volunteers. Differences in colonisation were highly significant (p<0.0001 in all cases; two sided Mann–Whitney test).

We first tested whether *H pylori* burdens were significantly lower in the so-defined UBT^−^ volunteer group. Indeed *H pylori* was reduced to ∼100 cfu/biopsy or undetectable, and thus was significantly different from the ∼10^4^ cfu/biopsy found in UBT^+^ volunteers (fig 2; p<0.0001; Mann–Whitney U test).

**Figure 3:**
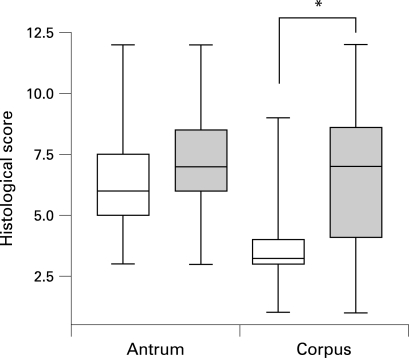
Development of antrum-predominant gastritis in [13C]urea breath test (UBT)-positive volunteers 3 months after challenge infection. Gastritis grade, activity, mucus depletion and regenerative changes were scored on a scale of 0–3. Data are represented in box and whisker plots which indicate median, range and 25th–75th centiles of the cumulative histological scores observed in UBT^−^ (shaded box) and UBT^+^ (open box) volunteers (cf. supplememtary tables 3 and 4). Histological scores of corpus gastritis were significantly different between the groups (*p<0.05; two-sided Mann–Whitney test).

We next compared histological parameters (supplementray tables 2 and 3) between UBT^−^ and UBT^+^ volunteers. Six weeks postchallenge, inflammation of the antrum and corpus showed no significant difference in extent or localisation between these groups. However, 3 months postchallenge, UBT^+^ volunteers had established an antrum-predominant inflammation pattern, a change not observed in the other volunteers (fig 3).

**Figure 4:**
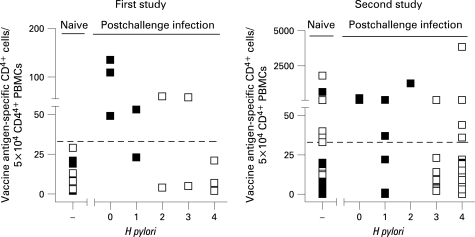
*H pylori*-specific CD4^+^ T cell response is negatively correlated with *H pylori* infection. Numbers of antigen-specific CD4^+^ T cells per 5×10^4^ CD4^+^ cells detected after short-term in vitro stimulation of 10^6^ peripheral blood monocytic cells (PBMCs) obtained before (denoted by naive) and 6 weeks after challenge infection (see supplementary table 6 for exact values) of urea breath test (UBT)*^−^* (▪) and UBT^+^ (□) volunteers were plotted against the overall *H pylori* score (tables 1 and 2). Stippled lines indicate thresholds (mean of prevaccination values + 3SD = 33 and 32 in the first and second study, respectively) for significant numbers in specific—that is, cytokine-producing, CD4^+^ T cells. In the left-hand panel (first study), *y*-values represent interferon γ (IFNγ)^+^, β7^+^, CD4^+^ T cell numbers responding against *H pylori* urease protein. In the right-hand panel (second study), *y*-values indicate β7^+^, CD40L^+^, interleukin2 (IL2)^+^ CD4^+^ T cell numbers responding to *H pylori* urease and/or HP0231 representing peptide pools. Fisher exact test p values that CD4^+^ T cell responder proportions were not similar in UBT*^−^* and UBT^+^ groups were: first study p = 0.103, second study p = 0.004, cumulated, p<0.001).

In animal models, reduction of bacterial burden requires specific T cells, and histological correlates such as altered inflammation have been described.^26^ As vaccine antigen-specific, circulating T cells were determined here (supplementary table 6), we tested whether these were correlated with reduced *H pylori* scores. Four of five volunteers (80%) in the first study and five of eight volunteers (62%) in the second study in the UBT*^−^* group showed significant *H pylori*-specific T cell responses 6 weeks postinfection (fig 4). In contrast, *H pylori*-specific T cells in PBMCs were detectable in only two of eight (25%; first study) and four of 37 (11%; second study) UBT^+^ volunteers. The negative correlation between T cell response and UBT positivity was significant in the second study, and was highly significant in the cumulated setting (Fisher exact test: first study p = 0.103, second study p = 0.004; if cumulated, p = 0.0002).

## DISCUSSION

Despite promise in preclinical models, the feasibility of vaccination against *H pylori* in humans remains unclear.^5^ In other cases of human-specific pathogens, experimental infection of volunteers for the development of vaccines has provided crucial insight.^27^^–^33 Here, we chose this approach to clarify the feasibility of vaccinating humans against *H pylori.* Like others,^34^ we critically evaluated this approach, and alternatives, such as therapeutic vaccination or field trials, were considered but would have had major drawbacks. For example, boosting non-protective, even pathogenic responses^35^ ^36^ in patients who may harbour treatment-resistant *H pylori* could not be excluded. The use of a defined challenge strain appeared to be advantageous in view of the extreme genetic variance of *H pylori* in patients.

Safety was of cardinal concern, and three vaccinated volunteers were challenged first to monitor clinical symptoms and tissue response. These were no different from those previously reported for non-vaccinated volunteers.^15^ Histological changes and the transcriptional profile recapitulated essential features of *H pylori* infection as known from patients,^17^ ^37^ and immediate resolution of inflammation even in clearing volunteers was not expected since gastritis also improves relatively slowly after antibiotic eradication.^15^ ^38^ With the caveat that our study protocols only allowed 3 months of observation, we conclude that experimental infection with *H pylori* enables vaccine testing in a controlled way.

Clearly, the Ty21a-derived vaccines were not satisfactory. Ty21a was used because it is a currently available, licensed human live vaccine and, although strongly attenuated,^39^ it is known to trigger mucosa-homing T and B cells.^24^ ^25^ ^40^ As a carrier, it induced but also boosted vaccine antigen-specific T cells expressing mucosa homing receptors, but not in all volunteers (this study, Bumann *et al*^16^ and Metzger *et al*^18^) which was suboptimal. Novel, more potent *S typhi* carriers show promise in overcoming this limitation.^41^ ^42^ The immunisation schedule may also be varied to improve induction of mucosa homing T cell responses. We noted a negative correlation between B cell (ASCs) and T cell responses against the carrier (*r*_sp_ = −0.4705; p = 0.0153). Official Ty21a vaccination schedules were adopted here, but these may favour B cell responses as protection against typhoid fever is positively correlated with the humoral response. Moreover, prime–boost regimens combining live and other vaccine formulations may be more potent, and therapeutic vaccination will also be worth exploring given the boosting effect noted here.

Importantly, we observed significant reduction of *H pylori* burden which was correlated with *H pylori*-specific T cell responses. This could have simply reflected passive loss of infection—“spontaneous clearance”—but in this case detecting correlations with two parameters (inflammation pattern and T cell response in peripheral blood) would not be expected. However, at present, the possibility that vaccination per se increased local innate and/or adaptive cross-reactive immune defence mechanisms leading to reduced *H pylori* burden cannot be excluded. In some volunteers, the origin of the T cells was unclear since they had no sign of active or serological evidence of past infection. In others, T cells were induced by vaccination.

*H pylori*-reactive T cells were not exlusively detected in UBT*^−^* volunteers, and it will be important to define markers of a protective response. In animal studies, despite numerous attempts,^14^ markers remain elusive. Results of transcriptome analyses in vaccinated mice have suggested unexpected mediators of immunity, adipokines,^43^ ^44^ but mechanisms are not understood. Interestingly, factorial analysis of the multidimensional cytometry data (not shown) from our T cell analysis suggests that qualitative differences in T cell responses detectable in PBMCs may exist between volunteers in UBT^+^ and UBT*^−^* groups. For example, CD40L^+^, IL4^+^ CD4^+^ T cells were predominantly associated with the T cell response in UBT^+ ^volunteers. Thus, defining predictive markers of protection may become possible.

Regulatory T cells may not be a hurdle for prophylactic vaccines as they were recruited into the inflamed stomach mucosa only at later time points, but may close a window of opportunity for therapeutic vaccination. Inversion of the challenge infection and vaccination protocol could help to address this important question in humans.

In summary, this is the first report to show that clearance of *H pylori* is correlated with an antigen-specific CD4^+^ T cell response, suggesting immunity against this infection in humans.
